# 608 Timing of Nanocrystalline Silver-based Dressing Application – a Retrospective Single-center Pediatric Cohort Study

**DOI:** 10.1093/jbcr/irae036.242

**Published:** 2024-04-17

**Authors:** Marshall Thibedeau, Julia Wenskus, Jennifer Zuccaro, Eduardo I Gus

**Affiliations:** University of Toronto, North York, ON; University children's hospital zurich, Zürich, Zurich; Hospital for Sick Children, Toronto, ON; SickKids / University of Toronto, Toronto, ON; University of Toronto, North York, ON; University children's hospital zurich, Zürich, Zurich; Hospital for Sick Children, Toronto, ON; SickKids / University of Toronto, Toronto, ON; University of Toronto, North York, ON; University children's hospital zurich, Zürich, Zurich; Hospital for Sick Children, Toronto, ON; SickKids / University of Toronto, Toronto, ON; University of Toronto, North York, ON; University children's hospital zurich, Zürich, Zurich; Hospital for Sick Children, Toronto, ON; SickKids / University of Toronto, Toronto, ON

## Abstract

**Introduction:**

Nanocrystalline silver-based dressings (NSBD) are commonly used at different stages of the treatment of burn wounds. They have been shown to reduce the incidence of surgical procedures, time to re-epithelialization in partial thickness burn injuries, and decrease pain with dressing changes compared to silver sulfadiazine in adult and pediatric populations. Recent evidence has demonstrated anti-inflammatory properties in addition to the known antimicrobial properties. We hypothesized that the timing of nanocrystalline silver-based dressings application ( < 24h after burn injury) may impact surgical treatment rates.

**Methods:**

This study was a retrospective single-center cohort study. All pediatric patients ( < 18 years old) treated at a single academic pediatric center for acute partial thickness burn injuries, between January 1, 2020 and December 31, 2021 were included. Bivariate analysis and multivariable logistic regression were used to compare timing of nanocrystalline silver application and surgical treatment rates.

**Results:**

Four hundred seventy-six patients were included for analysis. One hundred four (21.85%) had NSBD and 372 (78.11%) had non-silver non-adherent dressings applied within the first 24 hours post injury. No statistically significant difference was found in surgical treatment, age, sex, mechanism, anatomic location, or total body surface area (TBSA) on bivariate analysis. The multivariable logistic regression identified three statistically significant variables as predictors for surgical treatment: age (OR = 1.14, 95% CI [1.06-1.23]), TBSA (OR = 1.15, 95% CI [1.06-1.25]), and burns to buttocks/lower extremity burns (excluding feet) (OR = 2.39, 95% CI [1.26-4.53]). Immediate ( < 24h) application of NSBD has not been demonstrated to decrease the likelihood of surgical treatment.

**Conclusions:**

Immediate ( < 24h) application of nanocrystalline silver-based dressings does not affect surgical treatment rates. Prospective randomized studies are required to investigate the potential anti-inflammatory benefits of early nanocrystalline silver-based dressings in the treatment of pediatric burn injuries.

**Applicability of Research to Practice:**

Use of nanocrystalline silver should be recommended as per clinical indications.

